# Feasibility of Compliant Flooring in Long-Term Care: Results from a Stakeholder Symposium

**DOI:** 10.1017/S0714980817000551

**Published:** 2018-03

**Authors:** Chantelle C. Lachance, Dawn C. Mackey

**Affiliations:** 1 Department of Biomedical Physiology and Kinesiology, Faculty of Science, Simon Fraser University, British Columbia; 2 Knowledge Translation Program, Li Ka Shing Knowledge Institute, St. Michael’s Hospital, Ontario; 3 Centre for Hip Health and Mobility, Faculty of Medicine, University of British Columbia, British Columbia

**Keywords:** aging, knowledge dissemination, compliant flooring, injury prevention, long-term care, falls, vieillissement, diffusion des connaissances, revêtement de sol compliant, prévention des blessures, soins de longue durée, chutes

## Abstract

Compliant flooring aims to prevent fall-related injuries among high-risk older adults in long-term care, but uptake of compliant flooring in this setting is limited. We hosted a one-day stakeholder symposium to identify advantages and disadvantages of implementing compliant flooring in long-term care and the most pressing directions for future research from the perspective of key stakeholders. Twenty-three stakeholders representing health care, industry, and research attended the symposium. Attendees believed the most important advantages of compliant flooring were reducing injuries in residents who have fallen, potential benefits to care staff, and potential increases in quality of life for residents. Attendees perceived the most significant disadvantages of compliant flooring were financial considerations, lack of research evidence, and challenges with installation. Attendees indicated a need for additional research on cost-effectiveness and clinical effectiveness. While stakeholders perceived compliant flooring to add value to long-term care, there are significant informational and financial barriers to uptake.

## RÉSUMÉ

Le revêtement de sol compliant vise à prévenir les blessures causées par les chutes chez les personnes âgées à risque en soins de longue durée, mais l’utilisation de ce type de revêtement dans ces milieux est encore limitée. Nous avons organisé un symposium d’une journée réunissant les parties prenantes afin d’identifier les avantages et les désavantages de l’installation d’un revêtement de sol compliant en soins de longue durée, ainsi que les questions de recherche future les plus pressantes selon les perspectives des parties prenantes clés. Vingt-trois parties prenantes du domaine de la santé, de l’industrie et de la recherche ont assisté au symposium. Les participants considéraient que les avantages les plus importants de ce revêtement étaient la réduction des blessures pour les résidents qui ont chuté auparavant, les avantages potentiels pour le personnel soignant, et la possibilité d’amélioration de la qualité de vie des résidents. Les désavantages perçus comprenaient des considérations financières, le manque de résultats de recherche concernant ce revêtement et les défis associés à l’installation. Les participants ont indiqué que davantage de recherches étaient nécessaires pour montrer le rapport coût-efficacité et l’efficacité clinique du revêtement de sol compliant. Les parties prenantes ont ainsi perçu que ce revêtement procurait une valeur ajoutée en soins de longue durée, mais ont aussi mis en évidence certaines barrières informationnelles et financières significatives liées à son adoption.

Falls and fall-related injuries among older adults are common and costly. Approximately 30 per cent of community-dwelling adults aged 65 years and older will fall each year with 10-15 per cent of these falls resulting in serious injury (Blake et al., 1988; Campbell, Borrie, & Spears, 1989; Tinetti, Doucette, Claus, & Marottoli, 1995; Tinetti, Speechley, & Ginter, 1988). In the long-term care (LTC) setting, 60 per cent of older adults fall each year, and rates of injury are two- to threefold higher than those among the community-dwelling population (Rubenstein, Josephson, & Robbins, 1994). The consequences of falls among older adults exert a large financial burden on the health care system, including annual direct costs of $3.4 billion in Canada (Parachute, 2015) and $34 billion in the United States (Centers for Disease Control and Prevention, 2015). Thus, there is an urgent demand to reduce the incidence and severity of fall-related injuries. Ninety-five per cent of hip fractures in older adults are due to falls (Norton, Butler, Robinson, Lee-Joe, & Campbell, 2000; Stevens & Olson, 2000). About 20 per cent of hip fracture patients die within one year of fracture, and approximately 50 per cent are unable to return home or live independently after being discharged from hospital (Norton et al., 2000; Stevens & Olson, 2000). Although age-adjusted rates of hip fracture have levelled recently, the rate of traumatic brain injuries due to falls has tripled over the past decade (Harvey & Close, 2012; Stevens & Olson, 2000). Traumatic brain injuries are now responsible for more than half of all fall-related deaths in older adults (Harvey & Close, 2012; Stevens & Olson, 2000). Survivors of fall-related traumatic brain injuries are at risk of increased morbidity and mortality and decreased quality of life (Chesnut et al., 1999).

Compliant flooring represents a unique intervention for fall injury prevention in settings where falls are common, such as LTC (which we define as homes for older adults where personal and nursing care is provided on a 24-hour basis [Korall et al., 2015]). Compliant flooring offers the potential to reduce the incidence and severity of fall-related injuries by decreasing the stiffness of the ground and the forces applied to the body parts that impact the ground (Wright & Laing, 2012). Accordingly, compliant flooring is an intervention specifically targeted at reducing the adverse consequences of fall events (i.e., injury prevention) rather than preventing falls from occurring. Compliant flooring has the potential to reduce the incidence and severity of fall-related injuries at all body sites that impact the ground. Furthermore, compliant flooring is a passive intervention, since its effectiveness does not rely on user adherence once it is installed (Lachance, Jurkowski, Dymarz, & Mackey, 2016; Wright & Laing, 2012).

Compared to hip protectors, exercise, and pharmaceuticals, compliant flooring is a newer intervention directed at fall injury prevention, and it has not yet been broadly implemented in LTC or other health care settings. Nevertheless, there is a considerable body of scientific evidence about compliant flooring, including studies on biomechanical efficacy (Laing & Robinovitch, 2009; Laing, Tootoonchi, Hulme, & Robinovitch, 2006; Wright & Laing, 2011), clinical effectiveness (Drahota et al., 2013; Gustavsson, Bonander, Andersson, & Nilson, 2015; Healey, 1994; Simpson et al., 2004), cost effectiveness (Latimer, Dixon, Drahota, & Severs, 2013; Zacker & Shea, 1998), and workplace safety (Lachance, Korall et al., 2016; Marras, Knapik, & Ferguson, 2009; Wynn, Riley, & Harris-Roberts, 2011). To facilitate the uptake and application of this evidence about compliant flooring in LTC settings, the *knowledge-to-action framework* (Graham et al., 2006; Straus, Tetroe, & Graham, 2009) underscores the importance of identifying relevant stakeholders, assessing the barriers and facilitators faced by stakeholders to using the relevant evidence, and tailoring research questions to address problems identified by stakeholders.

In the LTC setting, stakeholders from health care, research, and industry are involved in making decisions about fall injury prevention strategies, and past research on compliant flooring has engaged stakeholders from each of these sectors (Lachance, Jurkowski, et al., 2016; Lachance, Feldman, et al., 2016). However, there is limited understanding about stakeholder perceptions of the advantages and disadvantages to implementing compliant flooring and about the research questions that stakeholders deem most important to address in the future. To address these knowledge gaps, we hosted a stakeholder symposium with two primary objectives: (1) to identify the advantages and disadvantages of implementing compliant flooring in LTC from the perspective of a diverse group of key stakeholders, and (2) to identify the most pressing research gaps in the available evidence and related directions for future research on compliant flooring from the perspectives of these key stakeholders. Our secondary objective was to gather feedback about the usefulness of the stakeholder symposium format as a knowledge translation activity.

## Materials and Method

### Attendees and Study Design

We hosted a one-day stakeholder symposium at Fraser Health Authority Headquarters (Surrey, British Columbia) in September 2016. We recruited attendees, from our existing professional networks, to represent a broad audience of stakeholders from health care (e.g., LTC, acute care, regional health authorities), industry (e.g., flooring manufacturing, interior design), and research. We invited clinicians, allied health practitioners, researchers, interior designers, industry partners, health managers, and regulators. Attendees were not required to have any background knowledge or experience with compliant flooring.

The day began with a keynote address presented by an international expert in prevention of injury and disease. The talk focused on the use of environmental interventions to improve older adults’ mobility and functional independence, and prevent fall-related injuries. Following the keynote, content experts led a series of podium presentations to disseminate up-to-date evidence about compliant flooring on the following topics: (1) how compliant flooring works, including an overview of the mechanics; (2) the current available evidence related to compliant flooring, based on results from a scoping review; (3) the push forces required to use floor-based lifts over compliant flooring, based on results from an ergonomic evaluation; and (4) the perceived feasibility of compliant flooring from the perspectives of LTC senior management, based on results from an interview study. Each presentation was followed by a facilitated question and answer period.

Following the podium presentations, we led an interactive workshop to identify advantages and disadvantages of implementing compliant flooring in LTC, and to identify gaps in the available evidence and directions for future research about compliant flooring, from the perspectives of the symposium attendees. All attendees were invited to participate in the workshop and were considered equal contributors in all discussions. Attendees were classified based on their occupation into four broad stakeholder sub-groups: LTC management (directors, managers); clinical (medical, allied health professionals, LTC resident care coordinators); health authority (facility planners, consultants, managers); and research and industry (researchers, instructors, flooring industry representatives). Our intention was to provide an opportunity for related stakeholders to work together and to provide an environment where attendees would feel comfortable participating in the table discussions. Each table was set up to have five to six attendees and was moderated by a workshop facilitator to ensure everyone contributed. Workshop facilitators were trained to ask three key questions about compliant flooring in the LTC setting: (1) What do you believe are the advantages of having compliant flooring? (2) What do you believe are the potential disadvantages (main concerns) for implementing compliant flooring? (3) What other information would be useful to you (i.e., identify key gaps in the research evidence)?

To help ensure that everyone contributed to the session, attendees were asked to record their answers to each question on sticky notes, from their own perspectives and experiences based on their job position. Attendees were encouraged to write down as many advantages, disadvantages, and gaps as they could (∼5 minutes per topic). All attendees received handouts of the podium presentation slides and a plain-language summary of the existing compliant flooring evidence; they could refer to these materials as they worked. The sticky notes for each of the three topics were collected by the facilitator and displayed and organized by theme on a poster board so that the group could all see. Each group then collectively ranked their top three advantages, disadvantages, and gaps, and one attendee from each table presented their group’s top three selections to all attendees as part of a closing discussion section of the workshop. All sticky notes from the workshop session were retained; each group had different colour sticky notes so we could later identify which concepts came from each group. We concluded the day by having attendees complete an event evaluation form.

### Data Collection

We collected data before the symposium, during the workshop portion of the symposium, and at the end of the symposium. Leading up to the symposium, we emailed all attendees to obtain demographic information using a pre-event form (see online supplementary file #1). We asked attendees to indicate their job title, place of work, how their job or place of work is involved in preventing injuries among older adults, and why they chose to attend the symposium. During the symposium, we retained all sticky notes from the workshop and also recorded each group’s top three selections of advantages, disadvantages, and research gaps. At the end of the symposium we asked each attendee to complete a two-page post-event evaluation form (see online supplementary file #2), informed by Wathen, Sibbald, Jack, and Macmillan (2011). This self-administered post-event form asked attendees to provide additional demographic information and rate their perceptions of the symposium, including overall usefulness, to evaluate outcomes of our knowledge translation strategy (Graham et al., 2006). We also asked questions related to behaviour change as a way to monitor anticipated knowledge use (Graham et al., 2006). Finally, we asked each attendee to list what they considered to be the biggest advantage, disadvantage, and research gap related to compliant flooring; this was a member-checking strategy (Krefting, 1991) to ensure the data obtained from the workshop included the major opinions of all attendees.

### Data Analysis

We used JMP 12.0.1 software (SAS Institute) to calculate all descriptive statistics from the pre-event and post-event forms and NVivo 11.2.2 software (QSR International) to code and manage all long-form data obtained from the pre-symposium data collection, workshop, and event evaluation form.

The data from the sticky notes were considered the main data to inform our results. The lead analyst used a thematic approach (Braun & Clarke, 2006; Patton, 2002) by first developing initial codes from all individual sticky notes collected from the workshop (*n* = 209). The analyst then refined these codes to form themes and subthemes that were used to develop a thematic framework (Braun & Clarke, 2006; Moore et al., 2014). The themes and subthemes were then compared with the top three advantages, disadvantages, and research gaps identified collectively by each group during the workshop and by each participant based on their responses on the post-event evaluation forms. This analysis step was performed to ensure the top-ranked advantages, disadvantages, and research gaps identified by the groups were captured in the framework, as a way of member checking. Due to the format of the workshop, all themes were discussed by all workshop groups. In order for a code to be considered a subtheme, at least one workshop group had to classify it within their top three ranked advantages, disadvantages, and research gaps. Subthemes were then ranked based on their identified importance by the workshop groups (i.e., injury prevention was ranked as number one for all groups which became the top ranked subtheme; benefits to care staff was ranked as number two for three groups which became the second ranked subtheme, etc.). Examples of our coding scheme are presented in Table 1.

**Table 1:** Examples of the coding scheme used in thematic data analysis

Main Theme	Subtheme Code	Description of Subtheme Code	Examples from Participants
Advantages of implementing compliant flooring	Injury prevention	• Reduced incidence of injuries • Reduced severity of injuries	“…makes the environment safer for ALL residents.” – *Clinical group*
Disadvantages of implementing compliant flooring	Financial considerations	• Cost of flooring • Availability of funding for flooring and additional equipment requirements	“…requires expensive equipment to move on the floor” – *LTC management group*
Research gaps about compliant flooring	Uncertainties about cost-effectiveness	• Cost-analysis/cost-benefit/cost-effectiveness/total cost/cost model • Cost assessment of direct and indirect costs • Determine return on investment	“cost-effective[ness] findings typically based on hip fracture prevention …what about other injuries?” – *Research and industry group*

## Results

### Demographics of Attendees

Twenty-three stakeholders attended the symposium. Of these attendees, 23 (100%) completed the pre-event form, 23 (100%) attended the morning keynote and podium presentations, 17 (73.9%) attended the afternoon workshop, and 21 (91.3%) completed the post-event form. Six attendees were unable to attend the afternoon workshop because of work demands. Attendees had a mean age of 50.4 years (*SD* = 11.3 years; age range: 30–68 years) and 70.0 per cent were women (*n* = 16). Attendees primarily represented LTC (34.8%, *n* = 8), regional health authorities (26.1%, *n* = 6), research (17.4%, *n* = 4), industry (17.4%, *n* = 4), and acute care (4.3%, *n* = 1) (Table 2). Some attendees identified with more than one sector. Based on a self-reported, 5-point scale, attendees were relatively knowledgeable about fall and injury prevention strategies and compliant flooring before the symposium. All attendees worked in either British Columbia (87.0%, *n* = 20) or Ontario (13.0%, *n* = 3).

**Table 2:** Demographics of symposium attendees

Measure	*n* = 23
Age, years, mean (*SD*)	50.4 (11.3)
Women, *n* (%)	16 (70.0)
Sector, *n* (%)	
Long-term care	8 (34.8)
Health authority	6 (26.1)
Research	4 (17.4)
Industry	4 (17.4)
Acute care	1 (4.3)
Highest Level of Education, *n* (%)	
College diploma	2 (8.7)
Bachelor’s degree	8 (34.8)
Master’s degree	8 (34.8)
PhD	4 (17.4)
Medical degree	1 (4.3)
Years working in current position, mean (*SD*)	10.2 (9.2)
Previous involvement with researchers hosting the symposium, *n* (%)	
I was not aware of the research group until being invited to the symposium	2 (9.5)
I was aware of the research group but not much else	2 (9.5)
My colleague or someone I know had been involved in research projects with this research group	3 (14.3)
I had personally been involved in research projects with this research group	14 (66.7)
Previous knowledge of fall and injury prevention strategies, scale 1 (low) to 5 (high), mean (*SD*)	4.2 (1.1)
Previous knowledge of compliant flooring, scale 1 (low) to 5 (high), mean (*SD*)	3.6 (1.2)

*Note:* Data missing for 2 attendees for the following items: years working in current position, previous involvement with researchers hosting symposium, previous knowledge of fall and injury prevention strategies, and previous knowledge of compliant flooring.

### Main Themes

Three main themes – advantages, disadvantages, and research gaps – each with five subthemes, emerged from the workshop data (Table 3).

**Table 3:** Perceived advantages and disadvantages of implementing compliant flooring in LTC, and research gaps in the available evidence about compliant flooring that emerged from the data, ranked in order of importance

Main Themes	Subthemes
Advantages of implementing compliant flooring	1. Injury reduction 2. Benefits to care staff 3. Increased quality of life for residents 4. Potential health care savings 5. Improved perceptions of care home
Disadvantages of implementing compliant flooring	1. Financial considerations 2. Lack of research evidence 3. Installation challenges 4. Repercussions to care staff 5. General concerns about flooring performance
Research gaps about compliant flooring	1. Uncertainties about cost-effectiveness 2. Uncertainties about clinical effectiveness 3. Uncertainties about biomechanical efficacy 4. Uncertainties about flooring performance 5. Uncertainties about workplace safety

### Perceived Advantages of Implementing Compliant Flooring in LTC

Attendees identified several potential advantages associated with implementing compliant flooring in LTC (Table 3). Attendees believed the most important advantages of compliant flooring were reducing injuries in residents who have sustained a fall, the potential benefits to care staff, and the potential to increase the quality of life for residents. The group suggested that potential health care savings and improved perceptions of the care home, although of secondary importance, were additional advantages.

For the injury reduction subtheme, attendees highlighted that compliant flooring may reduce both the number and severity of fall-related injuries should a fall occur, including serious injuries like hip fractures and head injuries. Attendees believed compliant flooring may be superior to other injury prevention intervention strategies, such as hip protectors, as it has the ability to reduce injuries for any body part that impacts the ground by providing high force attenuation. Attendees also affirmed that compliant flooring enables the environment to be safer for all residents and may also reduce injuries from falls sustained by LTC staff and families and friends of the LTC residents who visit the care home.

Attendees perceived that compliant flooring may provide important benefits to care staff. If residents have fewer fall-related injuries following the implementation of compliant flooring, staff will likely experience reduced stress and workload (i.e., fewer injuries result in reduced paperwork and post-fall investigations), and will have more time to focus their energy on other quality issues. Attendees mentioned that compliant flooring may also reduce fatigue of the care staff when walking or standing on the flooring. Attendees suggested that compliant flooring may also help to stimulate the further development and use of technology (e.g., lifting equipment) to compensate for the increased forces required for care staff to maneuver equipment over compliant flooring. This is further described in the perceived disadvantages section below.

Symposium attendees also suggested that compliant flooring may improve the quality of life for residents. They stated this may occur, in part, as a direct downstream effect of injury reduction. Attendees also remarked that by having compliant flooring installed in LTC, residents (and their family members) may have an improved sense of security and safety, and residents may in turn increase their mobility and activity levels throughout the care home. Thus, residents may experience a decreased fear of falling and increased physical activity levels and independence. The group also suggested that compliant flooring may improve resident autonomy by replacing other interventions that residents and staff may not want to use (e.g., bedside mats that may cause tripping, hip protectors that residents do not want to wear, and pharmaceutical interventions).

Coinciding with a reduction in fall-related injuries, attendees discussed the important role that compliant flooring might play in reducing overall health care costs. This is based on the assumption that by reducing the number of serious injuries sustained by the residents, there will be a reduced number of hospital transfers and admissions, resulting in a reduction in health care dollars spent on fall-related injuries. In addition, if compliant flooring is found to reduce injuries, attendees proposed that care homes with compliant flooring may be viewed as more desirable by the public; one means of achieving this could be using this flooring as a marketing tool by advertising the site as an innovative and proactive care home.

### Perceived Disadvantages of Implementing Compliant Flooring in LTC

Attendees identified several potential disadvantages of implementing compliant flooring in LTC, classified into five subthemes. The biggest perceived disadvantages were financial considerations, lack of research evidence, and challenges with installation (i.e., renovation of existing LTC sites). Of slightly less importance, although still of concern, were negative repercussions involving staff and general concerns about flooring performance.

Attendees ranked cost as the number one disadvantage associated with compliant flooring. Cost was described in a multitude of ways, including the cost of the material itself (relative to standard flooring), installation, maintenance, and additional equipment costs (e.g., purchasing motor-driven floor-based lifts to replace conventional floor-based lifts) to account for the differences in flooring stiffness versus standard flooring. Attendees were also concerned with who would provide the LTC care sites with the funding and how the costs of the flooring could be justified.

Collectively, attendees believed that the lack of research was a disadvantage. Attendees believed that more research needs to be performed before considering widespread implementation of compliant flooring in LTC. Specific examples of perceived unknowns include effects on balance, long-term utility (i.e., how well it works in real life), and clinical effectiveness.

The attendees remarked that installing compliant flooring in an existing care home could present significant challenges. It would be disruptive for residents and staff. If a care home decided to renovate only a portion of the total floor surface, the need would arise for installation of transitional ramps to account for height differences between the standard flooring and the compliant flooring system. Some of the attendees commented from personal experiences that these transitional areas can make it more difficult for residents to walk (with and without mobility aides) and may increase the risk of tripping for both residents and staff.

The attendees voiced concern that the implementation of compliant flooring in LTC may have potential repercussions for care staff. Specifically, they were aware that a floor with a lower stiffness would increase the rolling load resistance when care staff push or pull equipment and would possibly increase the risk of care staff sustaining musculoskeletal injuries. In addition, attendees were uncertain if all staff would want to adopt this type of injury prevention strategy.

Attendees also brought up general concerns about flooring performance. Namely, attendees were apprehensive about its durability, maintenance requirements, and sustainability in comparison to standard flooring. In addition, attendees acknowledged that the flooring will only have the ability to protect body parts that impact the floor, and not body parts that may impact a wall or furniture before impacting the ground.

### Research Gaps in the Available Evidence

Attendees indicated they still had uncertainties about cost-effectiveness, clinical effectiveness, biomechanical efficacy, flooring performance, and workplace safety of compliant flooring. The need for additional knowledge on cost-effectiveness and clinical effectiveness received the most emphasis.

A commonly discussed topic during the workshop was the lack of available evidence related to the cost-effectiveness of compliant flooring. Attendees indicated they would like additional cost-benefit and/or cost-effectiveness analyses performed to help determine whether compliant flooring should be installed in LTC. Attendees suggested that future economic analyses should include potential cost savings resulting from prevention of other injuries in addition to hip fractures (e.g., head injuries, wrist fractures), since most cost analyses have been performed by considering only hip fractures (Lange, 2012; Latimer et al., 2013; Njogu & Brown, 2008; Ryen & Svensson, 2015; Zacker & Shea, 1998). Other ideas presented included the following: (1) performing cost assessments for both direct and indirect costs of injurious falls, (2) determining the financial life cycle of the product, and (3) determining the cost-effectiveness of compliant flooring in low-income environments when compared to standard flooring.

Attendees suggested the need, second to cost-effectiveness, for more research to determine the intervention’s clinical effectiveness in the form of longer (in duration) and/or larger (number of participants) randomized controlled trials. Attendees stated they would like to see more results from trials conducted with the population of interest (i.e., older adults in LTC) and multiple types of injuries (e.g., hip fractures, head injuries, and wrist fractures). Attendees also mentioned that it would be worthwhile to determine whether certain environments (e.g., adult day care facilities, acute care, LTC) or populations (e.g., stroke patients, dementia residents) would benefit more from compliant flooring than others. Attendees were also curious about whether compliant flooring would increase mobility and activity levels, decrease fear of falling, or increase the incidence of falls in LTC residents.

Attendees were interested to know more about the effects of compliant flooring on dynamic balance tasks and gait performance, including individuals that may have neurological deficiencies (e.g., stroke). Attendees also were interested in associations between compliant flooring and point loading (e.g., cane use) and non-vertical forces (e.g., rolling resistance of medical equipment). Furthermore, attendees also suggested there is an evidence gap on what types of equipment should be modified to ensure that the care staff are able to work safely over compliant flooring. Attendees mentioned the need to directly measure whether there is an increase in workplace injuries after installation of compliant flooring. They also discussed the need for manufacturers to optimize the “dual stiffness” characteristics of the flooring so that it is soft enough to reduce falls but rigid enough to not impair walking. Finally, attendees had general uncertainties about durability, hygienic properties, effect on the environment, and sustainability of available compliant flooring systems.

### Attendees’ Perceptions of Symposium

The majority of attendees ranked the symposium high in terms of its relevance to their current work (mean response 4.6 [*SD* = 0.7] points from a five-point scale), benefit of meeting colleagues and exchanging information about compliant flooring (4.7 [0.5] points), level of comprehension of the material presented (4.8 [0.4] points), overall quality of discussion and dialogue at the symposium (4.9 [0.4] points), and overall satisfaction with the symposium (4.9 [0.4] points; Table 4). All respondents stated they learned something by attending the symposium (100%, *n* = 21). Of these, 95.2 per cent (*n* = 20) stated they plan to share what they learned with others, and 42.9 per cent (*n* = 9) planned to change their behaviour.

**Table 4:** Attendees’ perceptions of the stakeholder symposium, on a 5-point scale, obtained from the post-event evaluation form

Variable	Mean	*SD*	Min	Max
Relevance to attendee’s current work	4.6	0.7	3	5
Benefit of meeting colleagues and exchanging information about compliant flooring	4.7	0.5	4	5
Level of comprehension of the material presented	4.8	0.4	4	5
Overall quality of discussion and dialogue at the symposium	4.9	0.4	4	5
Overall satisfaction with symposium	4.9	0.4	4	5

*Note:* Response categories ranged from 1 (low) to 5 (high); Responses based from 21 stakeholders; *SD* = standard deviation; Min = minimum; Max = maximum.

## General Discussion

Although a growing body of literature has suggested that compliant flooring may be a viable fall injury prevention strategy in LTC, little is known about the perceptions held by key stakeholders who are responsible for making decisions about fall injury prevention strategies. Guided by the knowledge-to-action framework (Graham et al., 2006; Straus et al., 2009), we conducted a one-day stakeholder symposium attended by 23 stakeholders representing health care, research, and industry. The majority of attendees were knowledgeable about fall and injury prevention strategies, including compliant flooring, prior to attending the symposium. We used an interactive workshop approach to obtain and rank attendees’ perceptions of the advantages and disadvantages of implementing compliant flooring and research gaps in the available evidence about compliant flooring in the LTC setting. We also asked attendees whether our selected knowledge translation activity, a stakeholder symposium, was worthwhile for them to attend.

Our findings suggest that while stakeholders perceive compliant flooring to potentially add value to the LTC setting, there are significant informational and financial barriers to realizing those benefits. There appeared to be general agreement on a range of advantages, disadvantages, and research gaps between the pre-assigned workshop groups.

The prevention of fall-related injuries in residents was ranked as the number one advantage for implementing compliant flooring, which is consistent with the overall purpose of compliant flooring systems (Wright & Laing, 2012). Wright and Laing (2012) emphasized that compliant flooring is an intervention approach that precludes the need for active user compliance and adherence (e.g., by residents or care staff) to ensure effectiveness, which is in contrast to hip protectors, exercise, and pharmacological agents. Similarly, symposium attendees also believed that a passive injury prevention strategy such as compliant flooring is a key advantage when considering a new intervention. Attendees advocated that compliant flooring may reduce fall injuries among individuals other than residents (i.e., staff, family, and visitors of residents). To our knowledge, this advantage has not been previously mentioned in the published literature, and it suggests that compliant flooring may be beneficial to individuals outside of the target user group. Furthermore, attendees ranked “benefits to care staff” as the second most important advantage of compliant flooring. Previous literature has already identified that compliant flooring may increase staff comfort during walking (Hanger, Hurley, Hurring, & White, 2014), but attendees provided additional insights of how it may benefit care staff. For example, if there is an overall decrease in fall-related injuries in the care home (by residents and others), care staff may experience reduced workload and lower stress levels. This is an important and previously undocumented advantage, as LTC care staff are subject to considerable work-related stress and report high levels of burnout (Woodhead, Northrop, & Edelstein, 2016).

Attendees ranked “increased quality of life” for residents as the third most important advantage. If compliant flooring were installed throughout a LTC site or in “hot spots” where falls occur very frequently, residents may feel safer, which may reduce their fear of falling and increase their mobility and activity levels thus improving their overall quality of life. To our knowledge, this has not been previously documented in the literature. Furthermore, attendees believed compliant flooring might improve resident autonomy by replacing interventions that residents and/or staff may not want to use. However, this perceived advantage for residents’ improved quality of life could also have negative consequences for the residents if sites decided to then not adhere to standard practice guidelines (e.g., stop using hip protectors).

When considering the drawbacks, financial considerations were ranked by attendees as the number one disadvantage. It has been previously documented that compliant flooring costs more than standard flooring (Laing & Robinovitch, 2009; Lange, 2012; Latimer et al., 2013; Njogu & Brown, 2008; Ryen & Svensson, 2015; Zacker & Shea, 1998). However, there was discussion at the symposium about the complexities of providing a business case for compliant flooring in Canada: the potential benefits of compliant flooring are realized as health care savings by the government, yet currently the implementation decision and expense is left to individual LTC sites. Thus, it may be hard for LTC sites in Canada to implement compliant flooring, as most do not have the funding or resources to install the flooring on their own, and they will not directly realize any cost savings provided by the flooring. Second, some attendees believed there were too many unknowns to consider implementing compliant flooring at this time. This concern overlaps with research gaps, which are further elaborated on below. Third, attendees believed installing compliant flooring in an existing building would be particularly challenging. Those who had previous experience with a retrofit installation voiced that a successful renovation requires considerable planning and support from LTC administration, front-line (care) staff, maintenance staff, and residents’ family members. In addition, because retrofit installations require significant time and money to complete, the more prepared the care home is, the less disruptive it would be to its residents.

Of the several research gaps identified by attendees, most emphasis was placed on the uncertainties around cost-effectiveness and clinical effectiveness. Though the symposium included a summary presentation of the available cost-effectiveness evidence, the amount of information available in the literature was unsatisfactory for attendees. Thus, to expand on the available literature, more research is warranted to determine the conditions under which specific types of compliant flooring are cost-effective, especially when considering all injuries avoided versus only hip fractures. In addition, further research establishing the setting-specific clinical and financial impacts would help clarify the business case for compliant flooring in LTC. Attendees were also dissatisfied with the amount of clinical evidence available and were hoping to hear about results from larger and longer randomized controlled trials from multiple settings (e.g., LTC, acute care). This demonstrates the need for more clinical trials to provide additional evidence about compliant flooring. Attendees also felt that future research should consider examining dynamic balance and gait performance over compliant flooring among end users as a lot of previous research was performed with young, healthy participants (Glinka, Cheema, Robinovitch, & Laing, 2013; Laing, Tootoonchi, Hulme, & Robinovitch, 2006; Soangra, Jones, & Lockhart, 2010; Soangra & Lockhart, 2012; Weaver & Laing, 2016). More testing should be performed with equipment and tasks that may pose workplace safety concerns for care staff to ensure that compliant flooring is implemented in ways that protect the safety of everyone exposed, not just residents.

Overall, we found the symposium format was useful for engaging with stakeholders. Attendees were satisfied with the format and found it to be valuable for the following reasons: (1) relevance to their current work, (2) meeting colleagues and exchanging information, (3) ease of understanding material presented, and (4) the quality of discussion and dialogue during the symposium.

### Limitations

We used a novel approach to explore the perceptions of key stakeholders about implementing compliant flooring in LTC. Although we included stakeholders from different professional backgrounds, some groups (e.g., LTC) were better represented than others (e.g., acute care). Attendees were invited from our existing networks in British Columbia and Ontario and, therefore, may have had different perceptions than those from other regions of the country, and may not have held the same views as stakeholders in similar roles from other countries. Moreover, the symposium did not directly address the perspectives of LTC residents and their families, an important stakeholder group for the successful implementation of compliant flooring in LTC. Future research would benefit from partnering with these groups. Though our sample size afforded meaningful engagement from all attendees during the workshop, it precluded the ability to stratify the results by subgroup. Finally, our approach focused on implementing compliant flooring within LTC. Therefore, the results may not translate directly to other settings, such as the community or acute care, though similar methods could be used to explore advantages, disadvantages, and research gaps in those settings.

## Conclusion

In conclusion, attendees identified key advantages and disadvantages of implementing compliant flooring, as well as important gaps in evidence about compliant flooring that should be prioritized by future studies. By attending the workshop, attendees gained awareness about compliant flooring systems for preventing fall-related injuries among residents and an understanding of the evidence supporting its use as a technology to prevent fall-related injuries. We anticipate that the results of this symposium will facilitate future research projects to expand knowledge on compliant flooring for injury prevention.

## Supplementary material

For supplementary material accompanying this paper visit http://dx.doi.org/10.1017/S0714980817000551. click here to view supplementary material

## References

[ref1] Blake A. J., Morgan K., Bendall M. J., Dallosso H., Ebrahim S. B. J., Arie T. H. D., … Bassey E. J. (1988). Falls by elderly people at home: Prevalence and associated factors. Age and Ageing, 17(6), 365–372. Retrieved from http://ageing.oxfordjournals.org/content/17/6/365.short 3266440 10.1093/ageing/17.6.365

[ref2] Braun V., & Clarke V. (2006). Using thematic analysis in psychology. Qualitative Research in Psychology, 3(2), 77–101. Retrieved from http://dx.doi.org/10.1191/1478088706qp063oa

[ref3] Campbell A. J., Borrie M. J., & Spears G. F. (1989). Risk factors for falls in a community-based prospective study of people 70 years and older. Journal of Gerontology, 44(5), M112–M117. doi:10.1093/geronj/44.5.M112 2738307 10.1093/geronj/44.4.m112

[ref4] Centers for Disease Control and Prevention. (2015). Web–based injury statistics query and reporting system (WISQARS). Atlanta, GA: National Center for Injury Prevention and Control.

[ref5] Chesnut R., Carney N., Maynard H., Patterson P., Clay Mann N., & Helfand M. (1999). Rehabilitation for traumatic brain injury. Rockville, MD: Agency for Health Care Policy and Research.

[ref6] Drahota A. K., Kward D., Udell J. E., Soilemezi D., Ogollah R., Higgins B., … Severs M. (2013). Pilot cluster randomised controlled trial of flooring to reduce injuries from falls in wards for older people. Age and Ageing, 42(5), 633–640. doi:10.1093/ageing/aft066 23868093 10.1093/ageing/aft067

[ref7] Glinka M. N., Cheema K. P., Robinovitch S. N., & Laing A. C. (2013). Quantification of the trade-off between force attenuation and balance impairment in the design of compliant safety floors. Journal of applied biomechanics, 29(5), 563–572. 23271146 10.1123/jab.29.5.563

[ref8] Graham I. D., Logan J., Harrison M. B., Straus S. E., Tetroe J., Caswell W., & Robinson N. (2006). Lost in knowledge translation: Time for a map? The Journal of Continuing Education in the Health Professions, 26(1), 13–24. doi:10.1002/chp.47 16557505 10.1002/chp.47

[ref9] Gustavsson J., Bonander C., Andersson R., & Nilson F. (2015). Investigating the fall-injury reducing effect of impact absorbing flooring among female nursing home residents: Initial results. Injury Prevention, 21(5), 320–332. Retrieved from http://www.diva-portal.org/smash/record.jsf?pid=diva2%3A771163&dswid=5578 25833258 10.1136/injuryprev-2014-041468

[ref10] Hanger H. C., Hurley K., Hurring S., & White A. (2014). Low Impact flooring – Is it practical in a hospital? In *Proceedings of the 6th Biennial Australia and New Zealand falls prevention conference, Sydney, Australia* (p. 66).

[ref11] Harvey L., & Close J. (2012). Traumatic brain injury in older adults: Characteristics, causes and consequences. Injury, 43(11), 1821–1826. 22884759 10.1016/j.injury.2012.07.188

[ref12] Healey F. (1994). Does flooring type affect risk of injury in older in-patients? Nursing Times, 90(27), 40–41. 8047456

[ref13] Korall A. M. B., Feldman F., Scott V. J., Wasdell M., Gillan R., Ross D., … Lin L. (2015). Facilitators of and barriers to hip protector acceptance and adherence in long-term care facilities: A systematic review. Journal of the American Medical Directors Association, 16(3), 185–193. doi:10.1016/j.jamda.2014.12.004 25704127 10.1016/j.jamda.2014.12.004

[ref14] Krefting L. (1991). Rigor in qualitative research: The assessment of trustworthiness, American Journal of Occupational Therapy, 45(3), 214–222. 2031523 10.5014/ajot.45.3.214

[ref15] Lachance C. C., Feldman F., Laing A. C., Leung P.-M., Robinovitch S. N., & Mackey D. C. (2016). Study protocol for the flooring for injury prevention (FLIP) study: A randomised controlled trial in long-term care. Injury Prevention, 22(6), 453–460. doi:10.1136/injuryprev-2016-042008 27044272 10.1136/injuryprev-2016-042008

[ref16] Lachance C. C., Jurkowski M. P., Dymarz A. C., & Mackey D. C. (2016). Compliant flooring to prevent fall-related injuries: A scoping review protocol. BMJ Open, 6(8), e011757. doi:10.1136/bmjopen-2016-011757 10.1136/bmjopen-2016-011757 PMC5013369 27531731

[ref17] Lachance C. C., Korall A. M. B., Russell C. M., Feldman F., Robinovitch S. N., & Mackey D. C. (2016). External hand forces exerted by long-term care staff to push floor-based lifts: Effects of flooring system and resident weight. Human Factors, 58(6), 927–943. doi:10.1177/0018720816644083 27098263 10.1177/0018720816644083

[ref18] Laing A. C., & Robinovitch S. N. (2009). Low stiffness floors can attenuate fall-related femoral impact forces by up to 50% without substantially impairing balance in older women. Accident Analysis & Prevention, 41(3), 642–50. doi:10.1016/j.aap.2009.03.001 19393817 10.1016/j.aap.2009.03.001

[ref19] Laing A. C., Tootoonchi I., Hulme P. A., & Robinovitch S. N. (2006). Effect of compliant flooring on impact force during falls on the hip. Journal of Orthopaedic Research, 24(7), 1405–1411. doi:10.1002/jor 16705716 10.1002/jor.20172

[ref20] Lange B. (2012). The impact of absorbent floor in reducing hip fractures: A cost-utility analysis among institutionalized elderly in Sweden [master’s thesis]. Retrieved from http://www.diva-portal.org/smash/get/diva2:537434/FULLTEXT01.pdf

[ref21] Latimer N., Dixon S., Drahota A. K., & Severs M. (2013). Cost–utility analysis of a shock-absorbing floor intervention to prevent injuries from falls in hospital wards for older people. Age Ageing, 42(5), 641–645. doi:10.1093/ageing/aft076 23838763 10.1093/ageing/aft076

[ref22] Marras W. S., Knapik G. G., & Ferguson S. (2009). Lumbar spine forces during manoeuvring of ceiling-based and floor-based patient transfer devices. Ergonomics, 52(3), 384–97. doi:10.1080/00140130802376075 19296324 10.1080/00140130802376075

[ref23] Moore J. E., Mascarenhas A., Marquez C., Almaawiy U., Chan W.-H., D’Souza J., … Straus S. E. (2014). Mapping barriers and intervention activities to behaviour change theory for Mobilization of Vulnerable Elders in Ontario (MOVE ON), a multi-site implementation intervention in acute care hospitals. Implementation Science, 9(1), 160. doi:10.1186/s13012-014-0160-6 25928538 10.1186/s13012-014-0160-6 PMC4225038

[ref24] Njogu F., & Brown P. (2008). Cost effectiveness of impact absorbent flooring in reducing fractures among institutionalized elderly. Auckland, NZL: School of Population Health, University of Auckland.

[ref25] Norton R., Butler M. E. G., Robinson E., Lee-Joe T., & Campbell A. J. (2000). Declines in physical functioning attributable to hip fracture among older people: A follow-up study of case-control participants. Disability and Rehabilitation, 22(8), 345–351. 10896094 10.1080/096382800296584

[ref26] Parachute. (2015). The cost of injury in Canada. Toronto, ON. Retrieved from http://www.oninjuryresources.ca/publications/item/cost-of-injury-in-canada-2015

[ref27] Patton M. Q. (2002). Qualitative research and evaluation methods. (3rd ed.). Thousand Oaks, CA: Sage.

[ref28] Rubenstein L. Z., Josephson K. R., & Robbins A. S. (1994). Falls in the nursing home. Annals of Internal Medicine, 121(6), 442–451. 8053619 10.7326/0003-4819-121-6-199409150-00009

[ref29] Ryen L., & Svensson M. (2015). Modelling the cost-effectiveness of impact-absorbing flooring in Swedish residential care facilities. The European Journal of Public Health, 26(3), 407–411. doi:10.1093/eurpub/ckv197 26498954 10.1093/eurpub/ckv197

[ref30] Simpson A. H. R. W., Lamb S., Roberts P. J., Gardner T. N., Evans J. G., & Grimley Evans J. (2004). Does the type of flooring affect the risk of hip fracture? Age and Ageing, 33(3), 242–246. doi:10.1093/ageing/afh071 15082428 10.1093/ageing/afh071

[ref31] Soangra R., Jones B., & Lockhart T. E. (2010). Effects of anti-fatigue flooring on gait parameters In Proceedings of the Human Factors and Ergonomics Society Annual Meeting, 54(23), 2019–2022. Los Angeles, CA: Sage. doi:10.1518/107118110X12829370264042

[ref32] Soangra R., & Lockhart T. E. (2012). Determination of stabilogram diffusion analysis coefficients and invariant density analysis parameters to understand postural stability associated with standing on anti-fatigue mats. Biomedical Sciences Instrumentation, 48(March), 415–422. Retrieved from http://www.pubmedcentral.nih.gov/articlerender.fcgi?artid=3716259&tool=pmcentrez&rendertype=abstract 22846314 PMC3716259

[ref33] Stevens J., & Olson S. (2000). Reducing falls and resulting hip fractures among older women. Home Care Provider, 5(4), 134–141. doi:10.1067/mhc.2000.109232 10931397 10.1067/mhc.2000.109232

[ref34] Straus S. E., Tetroe J., & Graham I. D. (2009). Defining knowledge translation review. Journal of the Canadian Medical Association, 181(3–4), 165–168. doi:10.1503/cmaj.081229 10.1503/cmaj.081229 PMC2717660 19620273

[ref35] Tinetti M., Doucette J., Claus E., & Marottoli R. (1995). Risk factors for serious injury during falls by older persons in the community. Journal of the American Geriatrics Society, 43(11), 1214–1221. 7594154 10.1111/j.1532-5415.1995.tb07396.x

[ref36] Tinetti M., Speechley M., & Ginter S. (1988). Risk factors for falls among elderly persons living in the community. The New England Journal of Medicine, 319(26), 1701–1707. doi:10.1056/NEJM198812293192604 3205267 10.1056/NEJM198812293192604

[ref37] Wathen C. N., Sibbald S. L., Jack S. M., & Macmillan H. L. (2011). Talk, trust and time: A longitudinal study evaluating knowledge translation and exchange processes for research on violence against women. Implementation Science, 6(1), 102. doi:10.1186/1748-5908-6-102 21896170 10.1186/1748-5908-6-102 PMC3178499

[ref38] Weaver T. B., & Laing A. C. (2016). The influence of safety flooring on reactive stepping. Oral presentation from the National Falls Prevention Conference, Calgary, AB.

[ref39] Woodhead E. L., Northrop L., & Edelstein B. (2016). Stress, social support, and burnout among long-term care nursing staff. Journal of Applied Gerontology, 35(1), 84–105. doi:10.1177/0733464814542465 25098251 10.1177/0733464814542465

[ref40] Wright A. D., & Laing A. C. (2011). The influence of novel compliant floors on balance control in elderly women—A biomechanical study. Accident Analysis & Prevention, 43(4), 1480–1487. doi:10.1016/j.aap.2011.02.028 21545881 10.1016/j.aap.2011.02.028 PMC3471987

[ref41] Wright A. D., & Laing A. C. (2012). The influence of headform orientation and flooring systems on impact dynamics during simulated fall-related head impacts. Medical Engineering & Physics, 34(8), 10–13. doi:10.1016/j.medengphy.2011.11.012 10.1016/j.medengphy.2011.11.012 22172523

[ref42] Wynn T., Riley D., & Harris-Roberts J. (2011) Ergonomics appraisal of the manual handling (push–pull) risk factors in areas using impact absorbing forces *(HuSU/11/13)*. Health and Safety Laboratory.

[ref43] Zacker C., & Shea D. (1998). An economic evaluation of energy-absorbing flooring to prevent hip fractures. International Journal of Technology Assessment in Health Care, 14(3), 446–57. doi:10.1017/S0266462300011429 9780531 10.1017/s0266462300011429

