# Supplementary material for: Feasibility of Compliant Flooring in Long-Term Care: Results from a Stakeholder Symposium
Source: Can J Aging. 2018 Mar;37(1):84–94. doi: 10.1017/S0714980817000551 (PMC5851049; doi:10.1017/S0714980817000551)
Supplement: Supplementary file 1 [file S0714980817000551sup001.zip › S0714980817000551sup001.docx]

**Pre-event Questions**

Name:

Job Title:

Professional designation, if applicable (e.g., PhD, BSc):

Place of work:

How is your job or place of work involved in preventing injuries for seniors (if applicable, <100 words):

Why did you choose to attend this symposium (<100 words)?

Anything else we should know?
