# Supplementary material for: Feasibility of Compliant Flooring in Long-Term Care: Results from a Stakeholder Symposium
Source: Can J Aging. 2018 Mar;37(1):84–94. doi: 10.1017/S0714980817000551 (PMC5851049; doi:10.1017/S0714980817000551)
Supplement: Supplementary file 1 [file S0714980817000551sup001.zip › S0714980817000551sup002.docx]

**Thank you very much for attending the Symposium! Your views are very important to us.**

**Please respond to the following questions. All information provided will remain anonymous.**

**Please answer the following questions by checking one box only for each question.**

**X**

| ***Scoring example*** | high 5 4 3 2 1 low |
| --- | --- |
| Before this symposium, my knowledge of fall and injury prevention strategies was …. | high 5 4 3 2 1 low |
| Before this symposium, my knowledge of compliant flooring was… | high 5 4 3 2 1 low |
| The relevance of the symposium for my current work was…. | high 5 4 3 2 1 low |
| The benefit of meeting colleagues and exchanging information about compliant flooring through the symposium was …. | high 5 4 3 2 1 low |
| My level of comprehension of the material presented today was… | high 5 4 3 2 1 low |
| Overall, the quality of discussion and dialogue of this event was… | high 5 4 3 2 1 low |
| Overall, my satisfaction with this event was…. | high 5 4 3 2 1 low |

Comments to help explain a particular score(s): ____________________________________________________ ______________________________________________________________________________________________________________________________________________________________________________________

**Please respond to the following questions by filling out the comment boxes. Answer each of the questions on behalf of yourself and the sector you best represent.**

What do you believe is the **biggest advantage for implementing compliant flooring** in healthcare systems (e.g., long-term care, acute care) with older adults at risk for falling?

What do you believe is the **biggest concern for implementing compliant flooring** in healthcare systems (e.g., long-term care, acute care) with older adults at risk for falling?

Please complete the back of the page.

The **next research project** of compliant flooring systems should examine….

**Please respond to the following questions by checking one box only for each question.**

After attending this all-day symposium, did you learn something? Yes No Don’t know

If yes,

Do you plan to share what you learned with others? Yes No Don’t know

Do you plan to change your behaviour? Yes No Don’t know

**Please complete the following demographic information below.**

| Age: _____________  Highest Level of Education Achieved: ___________________  Job Title: _________________________________________  Number of Years’ Experience in Current Position: _________ | Sex: ___________ |
| --- | --- |
| In your current position, do you believe you would be involved in decisions about whether to install (or not install) compliant flooring in a healthcare setting? | |
| What sector do you most identify with?   \| Long-term care  Acute care \| Health Authority  Interior Design/Construction \| Industry  Academia \| \| --- \| --- \| --- \| \| Other: _________________________________________________________ \| \| \| \| Before this symposium, how would you describe your previous involvement with the researchers at Simon Fraser University hosting this event (select all that apply)?  I was not aware of the research group until being invited to the symposium  I was aware of the research group but not much else  I had previously read literature/study findings from the research group  My colleague or someone I know had been involved in research projects with this research group  I had personally been involved in research projects with this research group \| \| \| | |

Please return the completed questionnaire to an event coordinator. Many thanks for your input!
